# Relation between *NOD2* genotype and changes in innate signaling in Crohn’s disease on mRNA and miRNA levels

**DOI:** 10.1038/s41525-016-0001-4

**Published:** 2017-02-08

**Authors:** Yun Chen, Mohammad Salem, Mette Boyd, Jette Bornholdt, Yuan Li, Mehmet Coskun, Jakob Benedict Seidelin, Albin Sandelin, Ole Haagen Nielsen

**Affiliations:** 1 The Bioinformatics Centre, Department of Biology and Biotech Research and Innovation Centre, University of Copenhagen, Ole Maaloes Vej 5, DK-2200 Copenhagen, Denmark; 2 Department of Gastroenterology, Medical Section, Herlev Hospital, University of Copenhagen, Herlev Ringvej 75, DK-2730 Herlev, Denmark

## Abstract

Crohn’s disease is associated with an altered innate immune response of pathogenic importance. This altered response can be associated to loss-of-function polymorphisms in the *NOD2* (nucleotide-binding oligomerization domain-containing protein 2) gene, but also changes in transcriptional and post-transcriptional regulatory layers, including microRNA activity. Here, we characterized the link between *NOD2* genotype and inflammatory-mediated changes in innate signaling by studying transcriptional and post-transcriptional activity in response to NOD2-agonist muramyl dipeptide in monocytes from healthy controls, and Crohn’s disease patients with and without *NOD2* loss-of-function polymorphisms. We measured the expression of genes and microRNAs in monocytes from these subjects after stimulation with muramyl dipeptide. Gene expression profiles mainly distinguished the actual muramyl dipeptide response, but not the genotype. A hyper-responsive phenotype was found in Crohn’s disease patients without *NOD2* mutations, characterized by upregulated cytokine receptors and general downregulation of microRNA expression. Conversely, microRNA expression could identify genotype-specific differences between subject groups but exhibited little change upon muramyl dipeptide treatment. Only two microRNAs showed muramyl dipeptide-induced response, including miR-155, which was found to regulate multiple genes and whose host gene was one of the highest muramyl dipeptide responders. miR-155 was upregulated in Crohn’s disease patients with *NOD2* mutations following lipopolysaccharide and *Escherichia coli* treatment, but the upregulation was substantially reduced upon muramyl dipeptide treatment. While Crohn’s disease patients with *NOD2* mutations on average showed a reduced muramyl dipeptide response, the cohort exhibited large individual variance: a small subset had inflammatory responses almost comparable to wild-type patients on both gene and miR-155 regulatory levels.

## Crohn’s disease: Genetics impacts molecular regulation of innate immunity on miRNA, but not gene level

The genetics of people with Crohn’s disease affects the molecular drivers of their dysregulated immune responses. Some individuals with Crohn’s harbor mutations in the NOD2 gene, which encodes a pathogen recognition receptor that binds to a molecule called muramyl dipeptide (MDP). To better understand how alternations in NOD2 can lead to increased susceptibility to gut inflammation, Yun Chen, Mohammad Salem and colleagues from the University of Copenhagen and Herlev Hospital, Denmark, analyzed the expression patterns of both genes and small, regulatory microRNAs in blood cells from healthy controls and from Crohn’s patients with and without NOD2 mutations. They exposed the cells to MDP, and saw that although gene acticity changed dramatically as a response, there was little difference between subjects, regardless of genetics. Conversely, microRNA expression showed genotype-specific differences that weren not impacted by MDP treatment. The findings underscore the importance of microRNAs in Crohn’s disease.

## Introduction

Crohn’s disease (CD)^1^ constitutes together with ulcerative colitis (UC)^2^ the two most prevailing disorders under the umbrella term inflammatory bowel disease (IBD).^3^ CD can affect any part of the gastrointestinal tract but most often the ileocecal region. General clinical manifestations of intermitting bowel inflammation are associated with abdominal pain, fever, prolonged diarrhea, and/or weight loss.^4^ In CD, genetic variations and environmental factors such as altered microbiota interact to produce the inflammatory background of the disease.^1, 5^ Along with changes in the adoptive immune system, an impaired innate immunity is believed to play a crucial role in the immunopathogenesis of CD.

Epidemiologic and linkage studies suggest that genetic factors play a clinical role in determining CD susceptibility.^6, 7^ One of the key genes identified through these studies is *NOD2* (nucleotide-binding oligomerization domain-containing protein 2),^8, 9^ encoding a cytosolic pathogen recognition receptor that specifically binds to muramyl dipeptide (MDP), the smallest bioactive component of peptidoglycans present in most bacteria.^10^ Activation of *NOD2* leads to induction of key immune signaling pathways involved in CD pathogenesis, including the upregulation of various pro-inflammatory cytokines.^11^ In addition, continuous MDP stimulation inhibits the development of experimental colitis in animal models,^12^ suggesting that normally functioning *NOD2* signaling pathways might be directly involved in protecting and maintaining the intestinal homeostasis. Furthermore, innate signaling is altered even in *NOD2* wild-type (WT) CD patients,^13^ implying post-transcriptional modifications of *NOD2* signaling pathways could be involved in re-establishment of normal innate responses, and this mechanism could be a future therapeutic target aiming at restoring microbial responses in CD. In this context, recent studies have linked *NOD2* to crucial post-transcriptional changes, including regulation by microRNAs (miRNAs) in different intestinal epithelial cell lines.^14–16^ miRNAs are short structured RNAs that play important roles in the regulation of gene transcription and translation in development and disease progression, including CD.^17, 18^ One of the most well-known miRNA regulators associated with inflammation and innate immunity is miR-155.^19–22^ Previous studies have shown an elevated level of miR-155 in colonic mucosa of CD and UC,^23, 24^ and depletion of miR-155 protects from experimental colitis in mice.^25^ Different pathogenic stimuli induce miR-155 in vitro, including viral infection^20, 26, 27^ and lipopolysaccharide (LPS).^28^ However, the role of miR-155 in immune cells in the context of CD and *NOD2* polymorphisms is not well established.

There are three major single-nucleotide polymorphisms (SNPs) of the *NOD2* gene (SNP8, SNP12, and SNP13) that are strongly associated to CD and cause a loss-of-function phenotype with a reduced response to MDP.^11^ SNP8 and SNP12 are missense mutations, whereas SNP13 is a frameshift mutation with the most drastic loss-of-function phenotype.^29^ The link between SNPs in *NOD2* and CD-associated inflammatory responses might have implications for the disease not only because an innate immune dysfunction is important for the pathogenesis of CD, but also since normalization of innate signaling alleviates colitis.^12, 30^ However, controversies exist on how alterations in *NOD2* signaling can lead to an increased susceptibility to inflammation, and what the role of post-transcriptional regulation in patients with/without disease-associated *NOD2* variants are for the resulting pathogenic innate immune response.^11^ Therefore, transcriptional profiles of *NOD2* mutant cells responding to MDP or bacterial stimulation have been analyzed using coverage-limited microarrays and focused either on mRNAs or miRNAs. The miRNA–mRNA regulation studies were either a single-target study^31^ or done in non-primary cells or immune cells derived from non-CD subjects.^15, 32^ Thus, it is important to investigate the interactions between miRNAs and genes genome-wide in patient-derived cells, and study how this is affected in innate immune response as function of different *NOD2* genotypes. To do this, it is necessary to investigate both RNA species from the same cells upon inflammation induction in cells with and without *NOD2* loss-of-function SNPs.

To this end, in this study we measured the expression of genes and miRNAs in human blood monocytes before and after MDP stimulation using genome-wide RNA and small RNA sequencing in an exploratory cohort consisting of healthy control subjects and CD patients with/without *NOD2* loss-of-function SNPs (*N* = 7 in total), and subsequently validated our major findings in larger patient groups (*N* = 29, in total) as well as all identified miRNA-gene interaction candidates in the THP-1 cell line (*N* = 8). Our data suggest that gene expression effectively identifies pathological MDP/*NOD2-*associated immune gene responses in blood monocytes from subjects without *NOD2* loss-of-function mutations. A pre-inflammatory, hyper-responsive phenotype was found in CD patients without *NOD2* loss-of-function SNPs. Conversely, the miRNA regulatory layer was surprisingly static before and after MDP stimulation, but could predict subject and genotype groups regardless of MDP treatment. Only two miRNAs, miR-190A and miR-155, responded to MDP. CD patients with *NOD2* loss-of-function SNPs were, as expected, on average less responsive to MDP compared to other subjects at both gene and miR-155 levels, but these patients displayed a surprisingly large variance in their responses ranging from a total loss to a nearly intact inflammatory response. Although *NOD2* loss-of-function SNP patients displayed reduced miR-155 induction after MDP treatment, miR-155 induction was unchanged when treating with LPS or intact bacteria treatment, suggesting that *NOD2* mutations only affect *NOD2*-pathway-elicited response and not other inflammatory pathways.

## Results

### Differential expression profiles between CD_WT_, CD_NOD2_, and control subjects

**Fig. 1:**
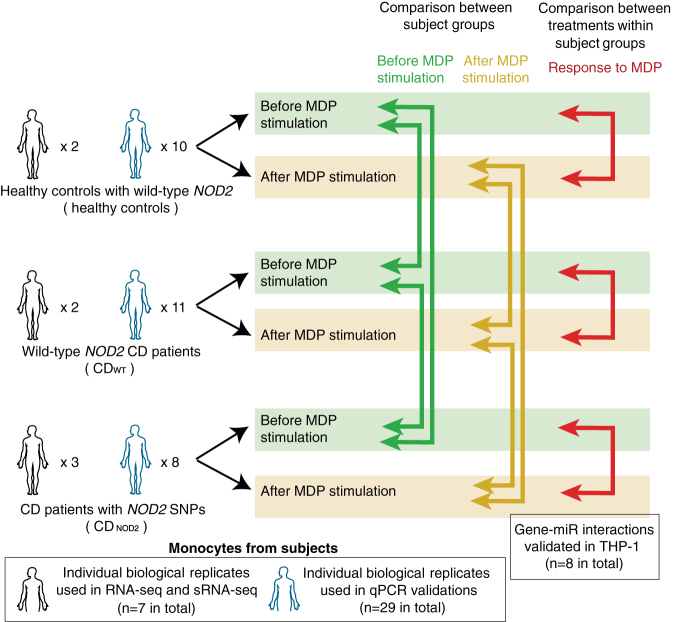
Overview of cohorts and differential expression analyses. Monocytes were derived from three subject groups: healthy controls, CD patients with WT *NOD2* genotypes (CD_WT_), and CD patients with loss-of-function *NOD2* genotypes (CD_NOD2_). Monocytes were analyzed by RNA-Seq and small RNA-Seq (*black* body outlines, number of tested individuals is indicated), and selected genes and miRNAs were validated by qPCR in a validation cohort (*blue* body outlines, number of tested individuals is indicated). We analyzed the difference between subject groups, before and after MDP stimulation (*green* and *yellow arrows*, shown in Figs. 2,3), and the response to MDP stimulation within each group (*red arrows*, shown in Fig. 4)

**Fig. 2:**
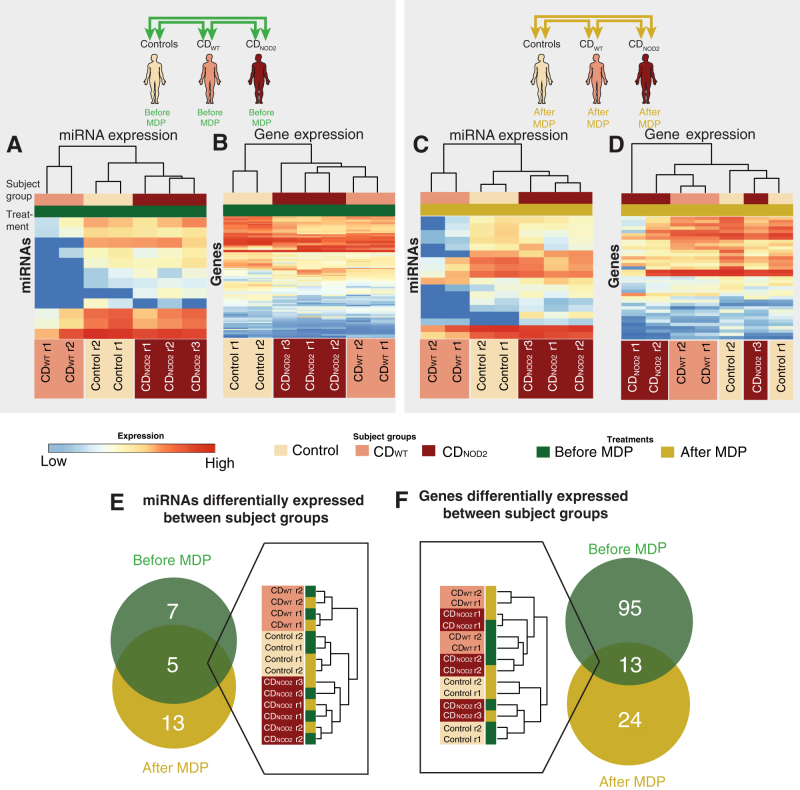
miRNA and gene expression patterns between subject groups. **a** Heat map showing the expression pattern of differentially expressed miRNAs between subject groups before MDP stimulation (*green arrows* in Fig. 1, also shown as schematics on *top*). Expression values are broken into permilles and assigned to a color gradient ranging from *red* (high expression) to *blue* (low expression). *Rows* indicate individual miRNAs, while *columns* show individual subjects. Rows and columns are ordered by hierarchical clustering using Euclidian distance, where the *top* dendrogram indicate similarity between subjects. Subject groups and MDP treatment are indicated by color above the heat maps. Note the clear separation between CD_WT_, CD_NOD2_, and controls. **b** As in **a**, but showing differentially expressed genes (by RNA-Seq) between subject groups before MDP treatment. **c** As in **a**, but showing differentially expressed miRNAs between subject groups after MDP stimulation. Note the clear separation between CD_WT_, CD_NOD2_, and controls. **d** As in **a**, but showing differentially expressed genes between subject groups after MDP treatment. **e** *Left*: Venn diagram showing the overlap between differentially expressed miRNAs between subject groups before and after MDP treatment. *Right*: expression-based clustering of the five shared miRNAs as in **a**, but only showing subjects, treatments, and the resulting dendrogram (color codes as in **a**–**d**). Note that subject groups are clearly separated, while MDP treatment status is not. **f** As in **e**, but for differentially expressed genes. Note that the shared gene set is not as efficient at distinguishing subject groups as the miRNA expression

We first analyzed the miRNA and gene expression signatures between the three subject groups: CD_WT_, CD_NOD2_, and controls using small RNA-Seq and RNA-Seq as defined in the “Methods” section before MDP treatment (green arrows, Fig. 1 and top of Fig. 2a,b) and after MDP treatment (yellow arrows, Fig. 1 and top of Fig. 2c,d).

Intriguingly, the miRNA expression signature could separate the three subject groups (controls, CD_NOD2_, and CD_WT_) both before and after MDP treatment. In both states, control subjects and CD_NOD2_ patients were more similar to each other than CD_WT_ patients, where many miRNAs were more lowly expressed compared to the other two groups (Fig. 2a,c). Thus, the overall miRNA population was downregulated in CD_WT_ patients, suggesting an overall lower impact of miRNA-mediated gene repression in this group.

In contrast, the differences of gene expression could not distinguish subject groups as clearly as the miRNA expression, because the differences between subject groups were much smaller than that observed by miRNA expression (see dendrogram in Fig. 2b,d, and Supplementary Fig. 1a, b).

We next analyzed the differentially expressed miRNAs and genes identified in these two analyses. Nearly half of miRNAs (42%, 5/12) that were differentially expressed between subject groups before MDP treatment were also differentially expressed between subject groups after MDP treatment (Fig. 2e, Venn diagram in the left panel). The expression levels of these shared miRNAs were highly consistent before and after MDP treatment (Supplementary Fig. 2a); thus, the overall regulatory layer of miRNAs seemed to be independent of MDP signaling between groups, and could at the same time separate the subject groups. Indeed, the five miRNAs that were differentially expressed between groups regardless of treatment could alone distinguish subject groups (but not MDP treatment) efficiently (Fig. 2e, dendrogram in the right panel).

**Fig. 3:**
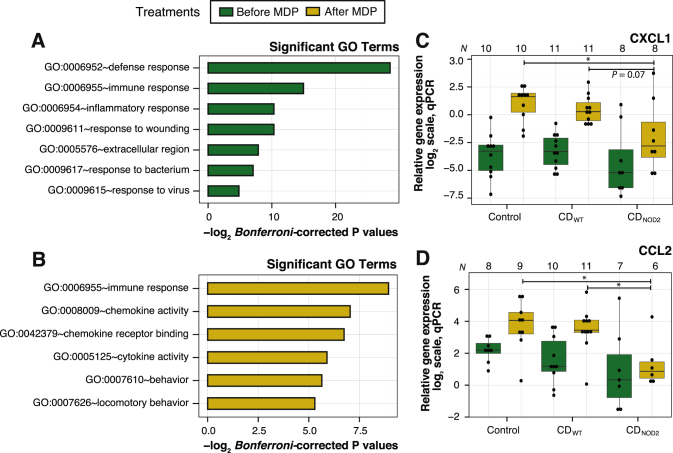
Functional annotation of differentially expressed genes between subject groups. **a** Enriched significant GO terms (*Bonferroni-corrected P-values* ≤ 0.05) for differential expressed genes between subject groups before MDP treatment. *X*-axis shows –log_2_(Bonferroni-corrected *P-*values) and *Y*-axis shows GO terms. **b** As in **a**, but showing significant GO terms for differential expressed genes between subject groups after MDP treatment. **c** qPCR validation for a selected chemokine, *CXCL1*, which exhibits a large difference between the subject groups after MDP treatment. *X*-axis shows subject groups before (*dark green*) and after MDP treatment (*yellow*). Numbers on *top* of the plot indicate the number of biological replicates passing the qPCR thresholds. Each subject is visualized as *black dots*. *Y*-axis shows the relative gene expression in log_2_ scale. *Asterisks* indicate level of significance (two-sided *t*-test): * indicates *P* ≤ 0.05, **P ≤ 0.01, and ***P ≤ 0.001. A single comparison with a *P*-value close to the significance threshold is indicated. **d** qPCR validation for a selected chemokine, *CCL2*, following the conventions of **c**

In contrast, only around 10% (13/108, Fig. 2f, also see Supplementary Fig. 1c) of genes that were significantly different between subject groups before MDP treatment were also significantly different between patient groups after MDP treatment. These shared genes could not separate the subject groups (Fig. 2f, dendrogram in the left panel). Consistently, these genes displayed a wide expression difference between subjects before and after MDP (Supplementary Fig. 2b). Genes that were differentially expressed between subject groups before MDP stimulation showed an enrichment of gene ontology (GO) terms relating to defense response, inflammatory response, response to wounding, and response to virus (Fig. 3a and Supplementary Fig. 3a). In contrast, genes differentially expressed between patient genotype groups after MDP treatment were enriched in chemokine and cytokine-related functions (Fig. 3b and Supplementary Fig. 3b). Quantitative PCR (qPCR) analysis for two selected chemokines, *CXCL1* and *CCL2*, in a larger cohort confirmed upregulation after MDP treatment in healthy controls and CD_WT_, but not in CD_NOD2_ patients (Fig. 3c,d; Supplementary Fig. 3c, d show corresponding RNA-Seq data). This is consistent with the established link between *NOD2* and cytokine induction through the NF-κB pathway.^11^

### miRNA and gene expression response to MDP

**Fig. 4:**
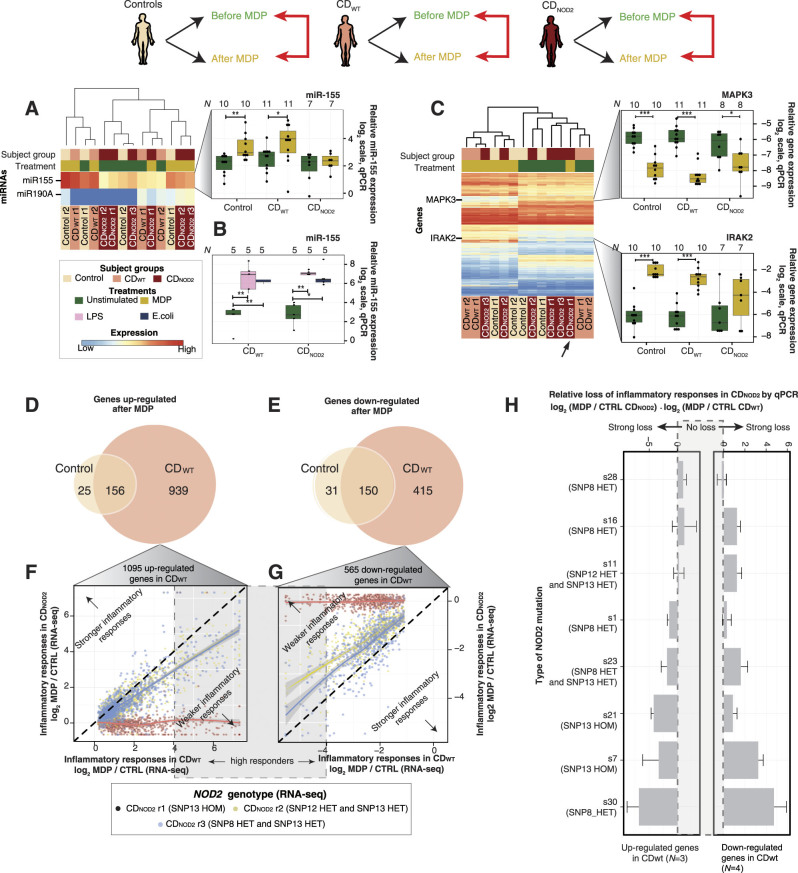
MDP-dependent miRNA and gene expression response within subject groups. **a** *Left*: Heat map as in Fig. 2a showing the two miRNAs that were differential expressed as a response to MDP within a subject group (schematic on *top* shows the analyzed comparisons). *Right*: qPCR validations for miR-155 are following the conventions of Fig. 3c. Asterisks indicate level of significance (two-sided paired *t*-test): * indicates *P* ≤ 0.05, **P ≤ 0.01, and ***P ≤ 0.001. **b** qPCR analysis of miRNA-155 expression LPS or intact *E. coli* in monocytes from CD patients, broken up by *NOD2* genotype and treatment. *Y*-axis shows the log_2_ normalized fold change of mir-155 vs. expression control (see Supplementary Methods for normalization): *dots* strand for individual patients. *Asterisks* indicate level of significance (two-sided paired *t*-test): * indicates *P* ≤ 0.05, **P ≤ 0.01, and ***P ≤ 0.001. **c** *Left*: As in *left* of **a**, but showing genes that were differential expressed as a response to MDP within a subject group. The *black arrow* highlights the outlier CD_NOD2_ subject that shows no substantial change in response to MDP. *Right*: qPCR validations of *IRAK2* and *MAPK3* genes are shown in the *right* panel, organized as in the *right* of panel a. **d** Venn diagrams showing the overlap of upregulated genes in response to MDP within subject groups. **e** Venn diagrams showing the overlap of downregulated genes in response to MDP within subject groups. **f** Degree of inflammatory gene response of individual CD_NOD2_ patients compared to the average response of CD_WT_ patients from the exploratory cohort. The inflammatory response is measured in a set of 1095 genes that were upregulated in CD_WT_. *X*-axis shows log_2_ fold change of the 1095 genes in CD_WT_ patients after MDP. *Y*-axis shows corresponding fold changes in individual CD_NOD2_ patients. Each *dot* corresponds to one gene, colored by individual CD_NOD2_ patients. *Solid lines* represent fitted linear models for each CD_NOD2_ patient using the loess approach; 95% confidence intervals are indicated as *gray* shadows. The *dashed* diagonal in *black* corresponds to equivalent inflammatory response between CD_NOD2_ and CD_WT_ patients. Genes located above the diagonal will correspond to stronger inflammatory response in a CD_NOD2_ patient than the average CD_WT_ response. Conversely, genes located below the diagonal will correspond to decreased inflammatory response in CD_NOD2_ vs. CD_WT_ patients. Genes with high log_2_ fold changes (>4) are considered high MDP responders (see main text). **g** As in **e**, but for the 565 downregulated genes in CD_WT_ patients following MDP stimulation. The two parts of the plot indicating stronger or weaker inflammatory response in a CD_NOD2_ are now swapped to the opposite. Genes with low log_2_ fold changes (<−4) are considered high MDP responders (see main text). **h** Relative loss of inflammatory response in individual CD_NOD2_ patients compared to average CD_WT_ patients in the validation cohort by qPCR. Each pair of horizontal *bar* plots shows the average degree of inflammatory response loss for a CD_NOD2_ patient, expressed as the average difference vs. CD_WT_ patients for three upregulated genes in CD_WT_ patients (*CXCL1*, *CCL2*, and *IRAK2*, *left* panel) and four downregulated genes in CD_WT_ patients (*MAPK3*, *PALD1*, *MAVS*, and *FLI1*, *right* panel). Error bars indicate the standard error of the mean

In order to investigate cell response to MDP, we compared the difference in miRNA and gene expression before vs. after MDP treatment within each patient group (red arrows in Fig. 1b and top of Fig. 4). Consistent with the analysis above, only two miRNAs (miR-155 and miR-190A) were significantly changed as a response to MDP in any pairwise comparison. Because miR-155 was much higher expressed than miR-190A (Fig. 4a, left panel), and was previously established as a key inflammatory regulator,^21, 22, 33^ we validated its expression change by qPCR in the larger validation cohort. The results showed that miR-155 was significantly upregulated in controls and CD_WT_, but not in CD_NOD2_ after MDP treatment (*P* ≤ 0.05, paired two-sided *t*-test) (Fig. 4a, right panel; Supplementary Fig. 4a shows corresponding small RNA-Seq data). Notably, there was no difference in miR-155 upregulation between CD_WT_ and CD_NOD2_ patients when cells were treated with either LPS or intact intestinal *Escherichia coli* bacteria (Fig. 4b). Thus, miR-155 could be upregulated by multiple inflammatory stimuli but only the MDP-triggered upregulation was dependent on the *NOD2* genotype. This makes sense, as *NOD2* is a specific sensor for MDP, while LPS and *E. coli* activate other *NOD2*-independent inflammatory pathways.

Conversely, the gene expression profiles were drastically different after MDP treatment (Fig. 4c, left panel and Supplementary Fig. 4b) and could distinguish treated vs. non-treated samples with the exception of one CD_NOD2_ sample (see black arrows in Fig. 4c and Supplementary Fig. 4b). qPCR analysis in the larger validation cohort confirmed the changes in selected differential expressed genes (Fig. 4c, right panel and Supplementary Fig. 4c–f). Notably, CD_WT_ patients had more than six times the number of upregulated genes than healthy controls (1095 vs. 181, Fig. 4d; also see Supplementary Fig. 4g). Moreover, the large majority of upregulated genes in healthy controls (86%, 156/181) were also upregulated in CD_WT_ patients. To test whether these 156 shared upregulated genes had the same magnitude of MDP response, we plotted the average gene expression fold change after MDP in the healthy control group vs. corresponding fold change in the CD_WT_ group (Supplementary Fig. 4h). In general, genes with high upregulation in the healthy control group had even higher upregulation in CD_WT_ patients. This was also true for the host gene of miR-155, consistent with the higher upregulation of mature miR-155 in CD_WT_ (Fig. 4a), and indicating an important role for miR-155 in MDP response in CD conditions.

Conversely, in the CD_NOD2_ patients, no genes were significantly upregulated or downregulated as a response to MDP (only ten genes were induced using the alternative significance calculation method, Supplementary Fig. 4g).

Together, these observations indicated that only subjects with WT *NOD2* (healthy controls and CD_WT_ patients) had substantial MDP-induced responses and CD_WT_ patients had a stronger and wider MDP response. To investigate the responses only found in CD_WT_ patients further, we mapped the shared upregulated genes between CD_WT_ and control subjects and the genes only upregulated in CD_WT_ subjects onto cytokine-receptor KEGG pathways.^34^ Strikingly, the receptors for upregulated cytokines were primarily upregulated in CD_WT_ patients but not in healthy controls (Supplementary Fig. 5a, b), suggesting that the downstream inflammatory response in these pathways was blocked or diminished in healthy controls even if the relevant cytokines were present. These results strongly imply that CD_WT_ patients had a wider inflammatory response including substantial activation of chemokine receptors and their downstream signaling pathways. Thus, these patients could be characterized as a phenotype with pre-inflammatory hyper-reactive innate response. An investigation of MDP-dependent downregulated genes showed that although the number of downregulated genes was smaller than upregulated genes, the results were consistent to the pattern observed above: on gene level, CD_WT_ patients had a stronger and wider MDP-mediated inflammatory response than healthy controls, while CD_NOD2_ patients typically lost the majority of the inflammatory response to MDP (Fig. 4e and Supplementary Fig. 4g, h).

While we observed no significant gene changes upon MDP stimulation in CD_NOD2_ patients, the MDP states of two CD_NOD2_ subjects could be distinguished using gene expression profiles (see dendrogram in Fig. 4c, left panel). Thus, we reasoned that subtle expression changes might exist, and that the type of *NOD2* mutation might influence the degree of expression change. To investigate this further, we focused on the genes found upregulated and downregulated upon MDP treatment in CD_WT_ (1095 and 565 genes, respectively). For each CD_NOD2_ sample we plotted the RNA-Seq gene expression fold change after MDP treatment vs. the average gene expression fold change after MDP in the CD_WT_ group (Fig. 4f,g, and Supplementary Fig. 4g, right panels). While this confirmed the overall lower inflammatory response in CD_NOD2_ samples vs. CD_WT_, it also showed clear differences in inflammatory response across the individual CD_NOD2_ samples. This was consistent between upregulated and downregulated genes and was particularly clear for genes with high fold changes. This indicated the loss of inflammatory responses in CD_NOD2_ patients was not binary but a gradient across individuals. To verify our observation in a larger cohort, we measured the expression of seven validated MDP-responsive genes in CD_WT_ (three upregulated genes: *CXCL1*, *CCL2*, and *IRAK2*; four downregulated genes: *MAPK3*, *MAVS*, *PALD1*, and *FLI1*) in eight CD_NOD2_ and eleven CD_WT_ samples before and after MDP treatment by qPCR (Fig. 4h). From this analysis, the large variance in inflammatory response was evident: some CD_NOD2_ patients showed virtually no inflammatory response, while some had inflammatory responses comparable to CD_WT_ subjects. Overall, in our set, CD_NOD2_ patients carrying SNP13 HOM had the lowest responses to inflammation induced by MDP (Fig. 4f–h and Supplementary Fig. 4g).

### miRNA-gene associations in MDP response

**Fig. 5:**
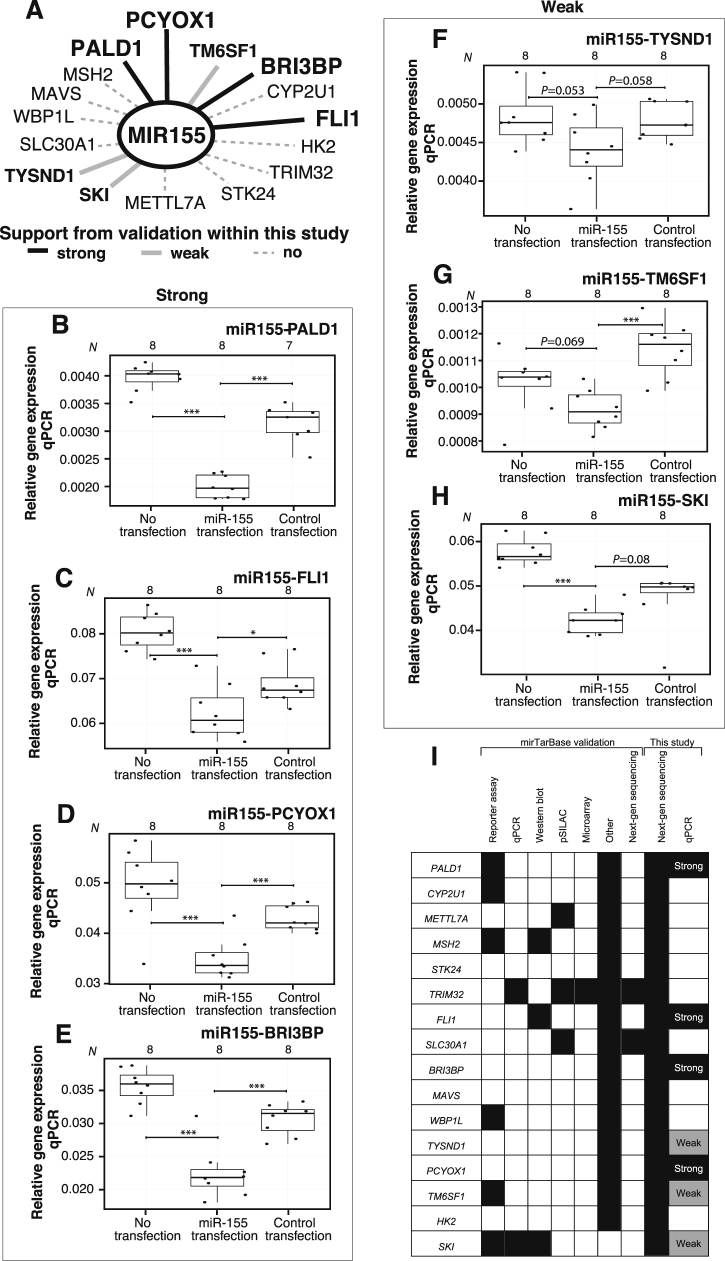
Identification of miR-155-gene associations. **a** miRNA-gene association network showing miR-155-gene interactions identified. *Line* color summarizes the qPCR-based transfection analysis results in the exploratory cohort (shown in **b**–**h**). Strong validations (*bold* lines, corresponding to **b**–**e**) and weak validations (*gray* lines, corresponding to **f**–**h**), and interactions that failed validations are shown as *dotted* lines. Strong and weak criteria are described in “Methods” (**b**–**h**). qPCR results of miR-155 transfected THP1 cells. *Y*-axis shows relative qPCR expression of the gene indicated on *top* of the plot. *X*-axis shows boxplots for cells that were not transfected, miR-155-transfected cells, and control-transfected cells. *Dots* indicate individual samples. *Asterisks* indicate level of significance (two-sided *t*-test): * indicates *P* ≤ 0.05, **P ≤ 0.01, and ***P ≤ 0.001. *P*-values for tests that are borderline significant are shown (see Methods). **b**–**e** show strong validation cases, **f**–**h** shows weak validation cases. **i** Summary of evidence for miR-155 regulation of the selected genes, from mirTarBase and this study. *Black* boxes indicate the evidence from the indicated type of experiments from mirTarBase. “Strong” and “weak” categories refer to the classification of validations in **b**–**h**

The analyses above did not take miRNA-elicited regulation of gene expression into account. To identify likely regulatory miRNA-gene associations, we used experimentally verified miRNA targets from mirTarBase 4.3,^35^ and required that the miRNA and gene pair in question had anti-correlated expression patterns (e.g., upregulation of miRNA and downregulation of gene, or vice versa) in any of the previously made pairwise expression analyses (any arrow in Fig. 1). When including all the differentially expressed miRNAs and genes, only 16 putative miRNA-gene pairs centered on miR-155 were identified (Fig. 5a). To further consolidate the regulatory role of miR-155, we transfected pre-miR-155 into human monocytic THP-1 cells and measured the expression change of the 16 candidate target genes with qPCR (Fig. 5a). Of these, *PALD1*, *FLI1*, *PCYOX1*, and *BRI3BP* were significantly repressed upon miR-155 transfection (*P* ≤ 0.05, two-sided *t*-test, Fig. 5b–e), while *TYSND1*, *TM6SF1*, and *SKI* were borderline significant (Fig. 5f–h, for details see “Methods”). It is important to consider that this type of experiment may include indirect effects; however, several of the genes had additional external evidence in the form of gene reporter assays (external evidence is summarized in Fig. 5i).

**Fig. 6:**
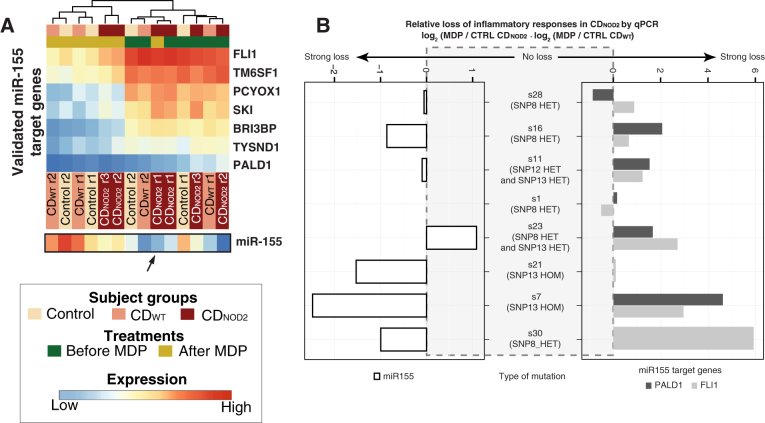
Classification power and variability of miR-155 and its targets across CD_NOD2_ patients. **a** Heat map as in Fig. 2a showing the expression pattern of the seven genes interacting with miR-155 indicated in **a**. The expression of miR-155 in corresponding libraries is indicated below the heat map. The *black arrow* highlights the outlier CD_NOD2_ subject that shows no substantial response to MDP. **b** Degree of response changes of individual CD_NOD2_ patients compared to the average response of CD_WT_ patients from the exploratory cohort on miR-155 level (*left* panel) and two selected miR-155 target genes (*PALD* and *FLI1*, *right* panel). The plot follows the same conventions as Fig. 4h

We found that this limited set of seven validated miR-155 targeting genes alone could distinguish MDP-treated cells from non-treated cells except for the same CD_NOD2_ subject that could not be correctly classified using the whole gene set (Fig. 6a). Because we identified a large variance across CD_NOD2_ patients on gene expression level following MDP stimulation, we hypothesized that a similar variation existed on miR-155 regulation level. In order to test this, we focused on miR-155 and the seven target genes identified above, and compared their MDP response between individual CD_NOD2_ patients and pooled CD_WT_ patients (Supplementary Fig. 6), similarly to our previous gene expression comparison in Fig. 4f,g. As expected, the CD_NOD2_ subject that showed the smallest inflammatory responses at gene level also displayed the most drastic loss of miR-155 upregulation and downregulation of its seven validated targets (Supplementary Fig. 6). This variance across patients was confirmed by qPCR analysis in the larger cohort. This showed that the loss of miR-155 induction following MDP treatment varied across subjects, and the corresponding level of downregulation of the miR-155 target genes generally followed the miR-155 levels (Fig. 6b). In general, CD_NOD2_ subjects displaying a reduced MDP response of gene level also showed this on miR-155 level (Figs. 4h, 6b).

## Discussion

In this study we explored gene and miRNA expression in an initial set of human blood monocytes using genome-wide high-throughput sequencing techniques and verified our major conclusions by applying qPCR analyses in a larger validation cohort or THP-1 cells. All the samples were split by MDP treatment and were broken up by subject groups, including patients with *NOD2* genotypes.

The results from pairwise differential expression studies between the subjects demonstrated that the MDP treatment resulted in considerable changes in gene expression. In particular, CD_WT_ patients showed a much stronger and wider inflammation response compared to healthy controls. This is in line with a report investigating the regulation of specific inflammatory pathways upon innate engagement of monocytes in CD,^36^ but we here show that these changes are more general, involving activating cytokine receptors and their downstream signaling pathways. In addition, it is noteworthy that while the major inflammatory responses in the gene layer of CD_WT_ patients corresponded to upregulation, miRNA expression was generally downregulated in these patients. This suggests that the innate hyper-responses upon inflammation in CD_WT_ patients might be also mediated by generally reduced activity of the miRNA regulatory layer. Such a *NOD2*-dependent phenotype of a pre-inflammatory innate hyper-responsiveness state could be essential in shaping the inflammatory processes found in CD. In line with this, there is evidence of an increased risk of onset of CD in patients after infections with intestinal pathogens, e.g., *Salmonella spp.* or *Campylobacter spp.* ^37, 38^

In contrast to mRNA levels, miRNA expression signatures showed large differences between subject groups, but only two miRNAs changed significantly with MDP treatment including a key inflammatory regulator, miR-155. Studies investigating *NOD2-*specific miR-155 inductions have shown MDP stimuli alone has limited impact on miR-155 expression in human dendritic cells,^15^ mouse macrophages,^27^ and embryonic fibroblasts.^39^ Our results demonstrated that MDP could induce miR-155 and its host gene in human monocytes expressing WT *NOD2*, and this induction was particularly strong in monocytes from CD patients, suggesting the *NOD2-*based induction on miR-155 might differ across both cell and disease types.

Previous studies have shown that the *NOD2* loss-of-function SNPs lead to decreased immune response,^40, 41^ but the differences between individual subjects and type of mutations were not addressed. Here, we showed that the effect of *NOD2* loss-of-function SNPs is diverse: some individuals from our CD_NOD2_ cohort lost the MDP response, while some could mount an inflammatory response similar to that of WT NOD2 subjects. This observation was reflected on both general gene expression profiles, on miR-155 levels and on its target genes. Patients carrying the SNP13 HOM appeared to have the largest response loss, although to investigate the detailed effect of respective genotype, expression quantitative trait loci analyses across a much larger population will be necessary.

As we have not induced or rescued *NOD2* mutations, these results are based on correlations between genotype and phenotype. However, as reduced induction of miR-155 in the CD_NOD2_ group occurred only when treating with MDP (a *NOD2* agonist) and not when treating with LPS or intact bacteria, it is highly likely that the *NOD2* mutations are the main drivers for the phenotypes observed and by extension, that inflammatory pathways not involving *NOD2* are likely intact in these patients. Additional mutations carried by these patients may modulate the MDP response and may explain the wide MDP-response repertoire in CD_NOD2_ patients. Such mutations may not only affect protein-coding genes but also regulatory sites, including the disruption of miRNA-binding sites, which have been reported to occur in immune-associated genes, including *NOD2* and *IRGM*.^42, 43^ However, to analyze such cases, genome-wide genotyping is necessary.

In summary, our study identified substantial and novel changes in *NOD2* signaling of CD_NOD2_ and CD_WT_ patients. The first group had on average dampened MDP responses, but the global response was highly varied across individuals, on both gene and miR-155 regulation levels. The CD_WT_ group was characterized by a pre-inflammatory innate hyper-responsive phenotype and thus responds much stronger to MDP than cells from healthy controls. This hyper-responsive phenotype was characterized by activation a wide range of cytokine receptors and potentially also by a general downregulation of miRNA expression in these patients. Both types of dysregulated innate responses could, in different ways, increase susceptibility to intestinal inflammation in response to commensal bacteria. Moreover, we have shed light on an underappreciated role of miRNAs in identifying disease-specific and genotype-specific alterations in CD.

## Methods

### Study population and genotyping

The patient population in this study was derived from a total of 57 healthy controls (i.e., without any known GI disorders) and 236 patients with well-established CD attending the IBD Clinic at Department of Gastroenterology, Medical Section, Herlev Hospital. The diagnostic criteria for CD were based on established guidelines^44^ applying clinical, radiologic, endoscopic, and histologic criteria. Screening for the common *NOD2* polymorphisms (i.e., SNP8 [Arg702Trp], SNP12 [Gly908Arg], and SNP13 [Leu1007fsinsC]) was performed as previously described.^45^ All participants in this study were WT in respect to *autophagy-related 16-like 1 (ATG16L1)* genotype. Patients included in the sequencing and validation studies were in complete clinical remission for at least 4 weeks prior to inclusion (Harvey-Bradshaw index ≤5),^46^ and did not receive glucocorticoids or biologics for at least 3 preceding months. Long-term treatment with thiopurines was, however, allowed if the dosing had been stable for more than 2 months. For RNA-Seq and small RNA-Seq, monocytes were isolated from peripheral blood samples from three subject groups (*N* = 7 in total): (I) healthy controls with WT *NOD2* (*N* = 2), (II) CD patients with WT *NOD2* (CD_WT_) (*N* = 2), and (III) CD patients with *NOD2* SNPs (CD_NOD2_; one SNP13 homozygote—replicate 1, one SNP13 heterozygote and SNP12 heterozygote—replicate 2, and one SNP13 heterozygote and SNP8 heterozygote—replicate 3) (*N* = 3). Subsequently, miRNA/mRNA patterns identified in the RNA-Seq exploratory experiments were tested in a larger cohort (*N* = 29 in total) where monocytes were obtained from (I) healthy controls with WT *NOD2* (*N* = 10), (II) CD_WT_ (*N* = 11), (III) CD_NOD2_ (mixed *NOD2* polymorphisms) (*N* = 8). The monocytes isolated from each participant were grown with or without MDP stimulation (see Supplementary Methods) for sequencing and qPCR analysis. Supplementary Table 1 shows additional subject data. Validations of miRNA-gene interactions were done with eight biological replicates in TPH-1 cells (see Supplementary Materials and Methods).

### Real-time qPCR validation of mRNAs and miRNAs

RNA was purified from monocytes and THP-1 cells, and converted to cDNA as described in Supplementary Methods. Primer sequences are shown in Supplementary Table 2. For each primer pair, only the samples that passed qPCR thresholds were used in respective analyses (see “Methods”). Numbers of samples passing thresholds are shown on top of corresponding figures. Two-sided *t*-tests qPCR values were used as default statistical tests between two groups. Paired tests were conducted for comparisons within subject groups shown in Fig. 4 and Supplementary Fig. 4. *P* ≤ 0.05 was used as significance cutoff. For miRNA transfection experiments in THP-1 cells, validations were considered “strong” if miR-155-transfected samples vs. no transfected samples and miR-155-transfected samples vs. control-transfected samples were both significant. Validations were considered “weak” either if *P* < 0.06 in both comparisons, or if *P* ≤ 0.05 in one of the two comparisons. Validations were considered “negative” if none of the above applied.

### RNA-Seq and small RNA-Seq preparation, mapping and quantification

Total RNA and short RNAs were purified using the Nucleospin RNA II isolation kit (Macherey-Nagel) as recommended by the manufacturer. Quantity and purity were determined on a NanoDrop1000, all samples had a 260/280 nm absorbance >1.9. Illumina libraries were constructed using TruSeq Stranded mRNA Sample Prep Kit and TruSeq Small RNA Library Preparation Kit (Illumina) following the manufacturer’s protocols. The libraries were sequenced on an Illumina Genome Analyser IIx running sequencing 76 cycles, single read, for the mRNA libraries, and 36 cycles, single read, for the short RNA libraries. Adapters were trimmed from reads, which were then, filtered by quality and mapped to the hg19 assembly (see Supplementary Methods).

### Differential expression analysis

Since our samples were derived from three subject groups and two MDP conditions, many types of pair-wise comparisons between groups were feasible, which would answer different types of questions (Fig. 1). Three group comparisons were performed as shown in Fig. 1: (1) between patient groups “before MDP treatment” (green arrows), (2) between subject groups “after MDP treatment” (yellow arrows), and (3) between expression profiles of the same subject group before and after treatment “MDP response” (red arrows).

For differential expression of genes in the above pair-wise comparisons, we conducted a combined analysis using DESeq^47^ and Cuffdiff,^48^ where we required an adjusted *P*-value ≤ 0.05 (Benjamini–Hochberg False Discovery Rate, FDR) from DESeq combined with a non-adjusted *P*-value ≤ 0.05 from Cuffdiff (shown in main figures). The reciprocal analysis requiring FDR ≤ 0.05 from Cuffdiff combined with a non-adjusted *P*-value ≤ 0.05 from DESeq did not give substantially different results (shown in Supplementary Figs. 1, 3–5). Differential expression analysis for miRNAs was performed by DESeq^47^ requiring a FDR ≤ 0.05. All differentially expressed genes analyzed with both methods and miRNAs are listed in Supplementary Table 3.

### Code availability

Aside from the programs mentioned above, computational analyses were performed in R. Code is available by request.

### Ethical approval

The study was approved by the Scientific Ethics Committee of the Copenhagen Capital Region. Written informed consents from all included subjects were obtained prior to participation, and the project fulfilled the Helsinki V Declaration.

## Electronic supplementary material

Supplementary Methods and Images

Supplementary Table 1

Supplementary Table 2

Supplementary Table 3
