# Supplementary material for: Relation between NOD2 genotype and changes in innate signaling in Crohn’s disease on mRNA and miRNA levels
Source: NPJ Genom Med. 2017 Feb 8;2:3. doi: 10.1038/s41525-016-0001-4 (PMC5642384; doi:10.1038/s41525-016-0001-4)
Supplement: Supplementary file 1 — Supplementary Methods and Images [file 41525_2016_1_MOESM1_ESM.pdf]

SUPPLEMENTARY METHODS AND IMAGES FOR

Relation between *NOD2* genotype and changes in innate signaling in Crohn's disease on mRNA and miRNA levels

Yun Chen[1,#], Mohammad Salem[2,#], Mette Boyd[1,&], Jette Bornholdt[1,&], Yuan Li[2], Mehmet Coskun[1,2], Jakob Benedict Seidelin[2,*], Albin Sandelin[1,*], and Ole Haagen Nielsen[2,*].
[1] The Bioinformatics Centre, Department of Biology and Biotech Research and Innovation Centre, University of Copenhagen, Denmark.
[2]Department of Gastroenterology, Medical Section, Herlev Hospital, University of Copenhagen, Denmark.
# Equal contribution
& Equal contribution
* Corresponding authors

SUPPLEMENTARY MATERIALS AND METHODS 2
SUPPLEMENTARY TABLE LEGENDS 7
SUPPLEMENTARY REFERENCES 8
SUPPLEMENTARY FIGURES WITH LEGENDS 10

SUPPLEMENTARY MATERIALS AND METHODS

Isolation and stimulation of monocytes

Peripheral blood mononuclear cells were separated from whole blood by Ficoll-Paque density gradient centrifugation according manufacturer's instructions (GE Healthcare). Monocytes were subsequently purified by negative selection using MACS beads (Miltenyi Biotech GmbH). After isolation, $1x10^6$ monocytes were plated in 24-well plates (NUNC Brand) in 950 µl growing medium (RPMI-1640 medium containing 10% FCS, 50 IU/ml penicillin, 50 µg/ml streptomycin, and 0.5 mg/ml gentamycin) at 37°C in an atmosphere of 5% CO_2 and a relative humidity of 90%. The monocytes rested overnight and were then stimulated with MDP (1 µg/ml), LPS (10 ng/ml), or *Escherichia coli* (at a ratio of 1 monocyte to 5 bacteria) for 4 hours, or cultured with vehicle (RPMI-1640) for 4 hours.

RNA purification from stimulated monocytes

For RNA-seq, small RNA-seq and validation analysis in monocytes, RNA were purified using the NucleoSpin®miRNA kit (Macherey-Nagel). The two step purification protocol was followed according to the manufacturer's instructions, with a final elution volume 40 ul. Small RNA (< 200 bp) and large RNA (> 200 bp) were isolated in two separate fractions.

Isolation, stimulation and transfection of THP-1 Cells

For pre-miRNAs transfection, THP-1 cells were cultured in a growing medium (RPMI-1640 medium containing 10% FCS, 50 IU/ml penicillin, 50 µg/ml streptomycin, and 0.5 mg/ml gentamycin) at 37°C in an atmosphere of 5% CO_2. The cells were transfected with pre-miRNAs (pre-miR-155, or control-miR) at a final concentration of 25 nM using Lipofectamin

RNAi MAX (Invitrogen). 8 biological-replicates samples for RNA analysis were harvested at 24-hours post-transfection.

Purification of total RNA from transfected and stimulated THP-1 cells.

For gene expression validation in THP-1 cells, total RNA was purified using PureLink® RNA Mini Kit (Ambion™, Life Technologies) combined with an on-column DNase treatment according to the manufacturer's instructions using lysis buffer added 1%β-mercaptoethanol and a final elution volume of 30 ul.

qPCR analysis of gene expression in monocytes and THP-1 cells

For validation of gene expression in monocytes and THP-1 cells, cDNA was synthesized using SuperScript III reverse transcriptase (Invitrogen) with a total input RNA of 200 ng for monocytes and 436 ng for THP-1 cells in a 20 µl reaction volume. All samples were run in triplicates in a 5 µl reaction volume consisting of 0.22 µl cDNA, 1.5 µl of primer mix (1.67 pmol/µl), 0.78 µl RNase-free water, and 2.5 µl Brilliant SYBR Green QPCR Master Mix (Invitrogen). PCR reactions were quantified using the QuantStudio 6 Flex Real-Time PCR System (Applied Biosystems). The thermal cycler details were as follows: 10 min at 95°C followed by amplification of the cDNA for 40 cycles with melting for 30 s at 95°C, annealing for 1 min at 60°C, and elongation for 45 s at 72°C.

qPCR data analysis of gene expression

The standard deviation (SD) between triplicates in qPCR experiments from both monocytes and THP-1 cells was required to be no more than 0.6. If the SD of triplicates exceeded 0.6, pairwise replicates were examined using the same threshold and the pairs

with the minimum SD were kept for further processes. The samples that did not satisfy these criteria in triplicates or in any of the replicate pairs were eliminated from the analyses. For the qPCR validation focusing on MDP responses using paired samples, only the samples satisfy these criteria in both 'before MDP' and 'after MDP' states were shown in the resulting plots. All results were normalized to human RPLP0 (ribosomal protein large p0) by the $2^{-\Delta Ct}$ method[1]. In order to eliminate batch differences between the two rounds of miR-155 experiments in monocytes using MDP stimuli (Figure 4A, right panel) and using LPS/E.coli stimuli (Figure 4B), for each unstimulated CD_{WT} and CD_{NOD2} subjects used in both experiments (5 for CD_{WT} and 4 for CD_{NOD2} subjects, for details see Supplementary Table 1), we calculated the qPCR-expression differences between experiments as $F_{batch} = \frac{Ustim_{Fig.4A}}{Ustim_{Fig.4B}}$ per subject and the median F_{batch} per subject groups was used for multiplying to the qPCR expression values derived from the later LPS/E.coli experiment.

qPCR analysis of miRNA expression in monocytes

For validation of miRNA expression, total RNA (10 ng) was reverse transcribed into cDNA using miRNA-specific primers (Applied Biosystems) and the TaqMan® miRNA reverse transcription kit (Applied Biosystems) according to the manufacturer's instructions. The expression levels of selected miRNAs were measured using commercially available pre-designed miRNA-specific TaqMan® miRNA assays (Applied Biosystems) according to the manufacturer's recommendations. PCR reactions were quantified using the LightCycler 480 real-time PCR system (Roche). All samples were run in triplicates in a 5 µl reaction volume and cycles were run as previously described by Coskun et *al.* [2]. The same SD threshold 0.6 as used in gene qPCR analysis was employed for examining technical triplicates or replicate pairs. Expression levels of each miRNA were normalized to human

RNU6B (RNA, U6B small nuclear). The relative expression levels of these miRNAs were determined by the $2^{-\Delta Ct}$ method[2].

Mapping and Quantification of RNA-seq libraries

After filtering the reads for a minimum sequencing quality of 30 in 50% of the bases with the FASTX-Toolkit (http://hannonlab.cshl.edu/fastx_toolkit/), nucleotides with quality score lower than 20 were subsequently trimmed from 3'end of the RNA-seq reads. The remaining reads were mapped to the hg19 genome assembly using Tophat v2.0.6[3] (settings: --segment-length 25) followed by a quantification of gene expression by Cufflinks2 v2.0.2[4] using Genecode version 15 as gene annotation. Before running differential expression analysis by DESeq[5] and Cuffdiff and to avoid the interference of non- and low expressed genes on the statistical model, genes with the lowest 40% expression (including genes with no expression) over all the libraries were discarded. Gene expression was visualized as FPKM (Fragments Per Kilobase of exon per Million mapped fragments) output from Cufflinks2.

Mapping and Quantification of Small RNA-Seq

After truncating the 3'-ends adaptors and filtering the reads for a minimum sequencing quality of 30 in 50% of the bases with the FASTX-Toolkit, we used Bowtie v0.12.7[6] (settings: --strata --best -k 10 -3 30) to map small RNA-Seq to the hg19 reference assembly. Because the protocol applied did not distinguish strands, miRNA levels were quantified by the sum of mapped reads falling into the corresponding genomic coordinates of their precursors using UCSC gene annotation, regardless of strand. The lowest 40% expressed miRNAs (including miRNAs with no expression) were removed before running

differential analysis in DESeq. The expression of miRNAs was visualized as TPM (tags per million mapped tags).

Association Between miRNAs and Their Potential Target Genes

We only analyzed miRNA-gene interactions supported by the published data in mirTarBase4.3[7]. We required miRNA and gene expression from each interaction to be reversely regulated in any of the differential expression pairs consisting of miRNAs and genes passing the cutoff from differential expression analysis (FDR ≤ 0.05) (arrows in Figure 1).

Gene Ontology Analysis and KEGG pathway analysis

GO analysis was performed using DAVID[8,9] using all expressed genes in all samples as background. GO terms (BP_FAT, CC_FAT and MF_FAT) were sorted by corresponding *Bonferroni*-adjusted *P* values. Gene enrichment analysis in KEGG[10] pathways was conducted using Pathview[11] using native KEGG graphs and default settings.

Pseudo counts and outlier truncation

For each analysis involving the use of log transformation when zero values existed in the analyzed dataset, the smallest non-zero value in the analyzed data points was employed as pseudo-count before log transformation. For the ease of visualization, in Figure 4F-H, Supplementary Figures 4G-H and 6, values smaller than the 1st or than the 99th percentile of all plotted values were truncated to 1st or 99th percentile.

SUPPLEMENTARY TABLE LEGENDS

Supplementary Table 1:

Patients characteristics

Supplementary Table 2:

Primer pairs used for gene expression validation.

Supplementary Table 3:

List of differentially expressed genes and miRNAs for respective group-wise analysis

SUPPLEMENTARY REFERENCES

1. Krzystek-Korpacka, M., Diakowska, D., Bania, J. & Gamian, A. Expression stability of common housekeeping genes is differently affected by bowel inflammation and cancer: implications for finding suitable normalizers for inflammatory bowel disease studies. *Inflammatory Bowel Diseases* **20,** 1147–1156 (2014).
2. Coskun, M., Bjerrum, J. T., Seidelin, J. B., Troelsen, J. T., Olsen, J. & Nielsen, O. H. miR-20b, miR-98, miR-125b-1*, and let-7e* as new potential diagnostic biomarkers in ulcerative colitis. *World J Gastroenterol* **19,** 4289–4299 (2013).
3. Kim, D., Pertea, G., Trapnell, C., Pimentel, H., Kelley, R. & Salzberg, S. L. TopHat2: accurate alignment of transcriptomes in the presence of insertions, deletions and gene fusions. *Genome Biol.* **14,** R36–R36 (2012).
4. Trapnell, C., Hendrickson, D. G., Sauvageau, M., Goff, L., Rinn, J. L. & Pachter, L. *Nat Biotechnol* **31,** 46–53 (2012).
5. Anders, S. & Huber, W. Differential expression analysis for sequence count data. *Genome Biol.* **11,** R106 (2010).
6. Langmead, B., Trapnell, C., Pop, M. & Salzberg, S. L. Ultrafast and memory-efficient alignment of short DNA sequences to the human genome. *Genome Biol.* **10,** R25 (2009).
7. Hsu, S.-D., Tseng, Y.-T., Shrestha, S., Lin, Y.-L., Khaleel, A., Chou, C.-H., Chu, C.-F., Huang, H., Lin, C.-M., Ho, S.-Y., Jian, T.-Y., Lin, F.-M., Chang, T.-H., Weng, S.-L., Liao, K.-W., Liao, I.-E., Liu, C.-C. & Huang, H.-D. miRTarBase update 2014: an information resource for experimentally validated miRNA-target interactions. *Nucleic Acids Research* **42,** D78–D85 (2013).
8. Huang, D. W., Sherman, B. T. & Lempicki, R. A. Systematic and integrative analysis

of large gene lists using DAVID bioinformatics resources. *Nat Protoc* **4,** 44–57 (2008).

9. Huang, D. W., Huang, D. W., Sherman, B. T., Sherman, B. T., Lempicki, R. A. & Lempicki, R. A. Bioinformatics enrichment tools: paths toward the comprehensive functional analysis of large gene lists. *Nucleic Acids Research* **37,** 1–13 (2009).

10. Kanehisa, M., Sato, Y., Kawashima, M., Furumichi, M. & Tanabe, M. KEGG as a reference resource for gene and protein annotation. *Nucleic Acids Research* **44,** D457–D462 (2016).

11. Luo, W., Luo, W., Brouwer, C. & Brouwer, C. Pathview: an R/Bioconductor package for pathway-based data integration and visualization. *Bioinformatics* **29,** 1830–1831 (2013).

Supplementary Figure S1 (expanding Figure 2):

This figure shows the same analyses as in Figure 2, but uses an alternative method for defining differentially expressed gene sets (FDR ≤ 0.05 from Cuffdiff combined with a non-adjusted P-value ≤ 0.05 from DESeq).

A) Heat map showing the expression pattern of differentially expressed genes between subject groups before MDP stimulation (green arrows in Figure 1, also shown as schematics on top). Expression values are broken into permilles and assigned to a color gradient ranging from red (high expression) to blue (low expression). Rows indicate individual miRNAs while columns show individual subjects. Rows and columns are ordered by hierarchical clustering using Euclidian distance. Subject groups and MDP treatment are indicated above the heat maps, as indicated by color.

B) As in A, but showing differentially expressed genes between subject groups after MDP treatment.

C) Venn diagram showing the overlap between differentially expressed genes across subject groups before and after MDP treatment.

Supplementary Figure S2 (expanding Figure 2):

A) Boxplots showing the TPM (Tag per Million) of 5 differential expressed miRNAs regardless of MDP treatment from Figure 2E. X-axis shows the subject groups while Y-axis shows the expression levels. Treatments are indicated by different colors.

B) As in A, but showing the RPKM (Reads Per Kilobase per Million mapped reads) of 13 differentially expressed genes regardless of MDP treatment (defined in Figure 2F).

Supplementary Figure S3 (expanding Figure 3):

Panels A and B show the same analyses as in Figure 3A-B, but use an alternative method for defining differentially expressed gene sets (FDR ≤ 0.05 from Cuffdiff combined with a non-adjusted P-value ≤ 0.05 from DESeq).

A) Enriched significant GO terms (*Bonferroni*-corrected *P values* ≤ 0.05) for differential expressed genes between subject groups before MDP treatment. X-axis shows –$\log_2$(*Bonferroni*-corrected *P* values) and Y-axis shows GO terms.

B) As A, but showing significant GO terms for differential expressed genes between subject groups after MDP treatment.

C-D) RNA-seq signals for the qPCR-validated chemokines (*CXCL1* and *CCL2*) shown in Figure 3C-D. Y-axis shows gene expression as RPKM. Error bars indicate standard error of the mean.

Supplementary Figure S4 (Corresponding to Figure 4):

Panels B and G show the same analyses as in Figure 4C-E, but use an alternative method for defining differentially expressed gene sets (FDR ≤ 0.05 from Cuffdiff combined with a non-adjusted P-value ≤ 0.05 from DESeq).

A) Corresponding small RNA-seq data for the validated miRNA-155 shown in Figure 4A (right panel), expressed as TPM. Error bars indicate standard error of the mean.

B) Heat maps as in Figure 4C showing genes that are differential expressed upon MDP treatment within a subject group.

C) As A, but showing RNA-seq data for the two validated genes shown in Figure 4C (right panel).

D) The left panel shows the expression of *MAVS* as measured by qPCR following the conventions in Figure 3C. The right panel shows the corresponding RNA-seq data as in A.

E) As D, but showing qPCR data and RNA-seq data for *PALD1.*

F) As D, but showing qPCR data and RNA-seq data for *FLI1.*

G) Left panel: Venn diagrams showing the overlap of up-regulated (top) and down-regulated (bottom) genes in response to MDP within subject groups. Right panel: Degree of inflammatory gene response of individual CD_{NOD2} patients compared to the average response of CD_{WT} patients from the exploratory cohort. The inflammatory response is measured in a set of 539 genes that were up-regulated in CD_{WT} and in a set of 240 down-regulated genes in $CD_{WT.}$ X-axis shows average log_2 fold change of these genes in CD_{WT} patients after MDP. Y-axis shows corresponding fold changes in individual CD_{NOD2} patients. Each dot corresponds to one gene, colored by individual CD_{NOD2} patients. Solid lines represent fitted linear models for each CD_{NOD2} patient using the loess approach; 95% confidence intervals are indicated as grey shadows. The dashed diagonal in black corresponds to equivalent MDP-mediated inflammatory response between CD_{NOD2} and CD_{WT} patients. The degree of inflammatory-response changes in SNP is indicated at corners with black arrows. Genes with high log2 fold changes (>4 or <-4) are considered high MDP responders.

H) Degree of MDP response of average healthy controls compared to the average response of CD_{WT} patients from the exploratory cohort. MDP response is measured in a set of 156 genes that were up-regulated in both healthy control and CD_{WT}, and in a set of 150 down-regulated genes in both healthy control and $CD_{WT.}$ X-axis shows average log_2

fold change of these genes in CD_{WT} patients after MDP. Y-axis shows corresponding average log_2 fold change in healthy controls. Each dot corresponds to one gene, colored by up- or down-regulation in response to MDP. Solid lines represent fitted linear models using the loess approach; 95% confidence intervals are indicated as colored shadows. The dashed diagonal in black corresponds to equivalent MDP-mediated inflammatory response between healthy controls and CD_{WT} patients. The degree of inflammatory-response enhancement in CD_{WT} patients is indicated at corresponding corners with black arrows. Genes with high log2 fold changes (>4 or <-4) are considered high MDP responders. The miR-155 host gene is highlighted.

Supplementary Figure S5 (expanding Figure 4):

A) KEGG "Cytokine-Cytokine Receptor" pathway map overlaid with MDP-induced up-regulated genes from different groups. Nodes, corresponding to the cytokines and their receptors, are colored based on the employed differential expression set (see Methods) and based on their up-regulation in subject groups or combinations of subject groups following MPD treatment, as indicated in the legend. Genes with no background are not differentially expressed. Note the high activation of cytokines in several of the subject groups, but the common upregulation of cytokine receptors in the CD_{WT}-only group.

B) As in panel A but for the KEGG "Chemokine Signaling" pathway. The same patterns as in A are evident: a general upregulation of the chemokine in both healthy controls and CD_{WT} subjects, but exclusive upregulation of downstream genes, including the chemokine receptors and NF-κB, a master regulator of inflammation response, in the CD_{WT}-only group.

Supplementary Figure S6 (Corresponding to Figure 6):

The figure follows the conventions of the right panel of Supplementary Figure 4G, but shows the degree of MDP response of individual CD_{NOD2} patients compared to the average response of CD_{WT} patients from the exploratory cohort, in terms of MDP-mediated expression changes of miR-155 and its seven validated targeting genes.

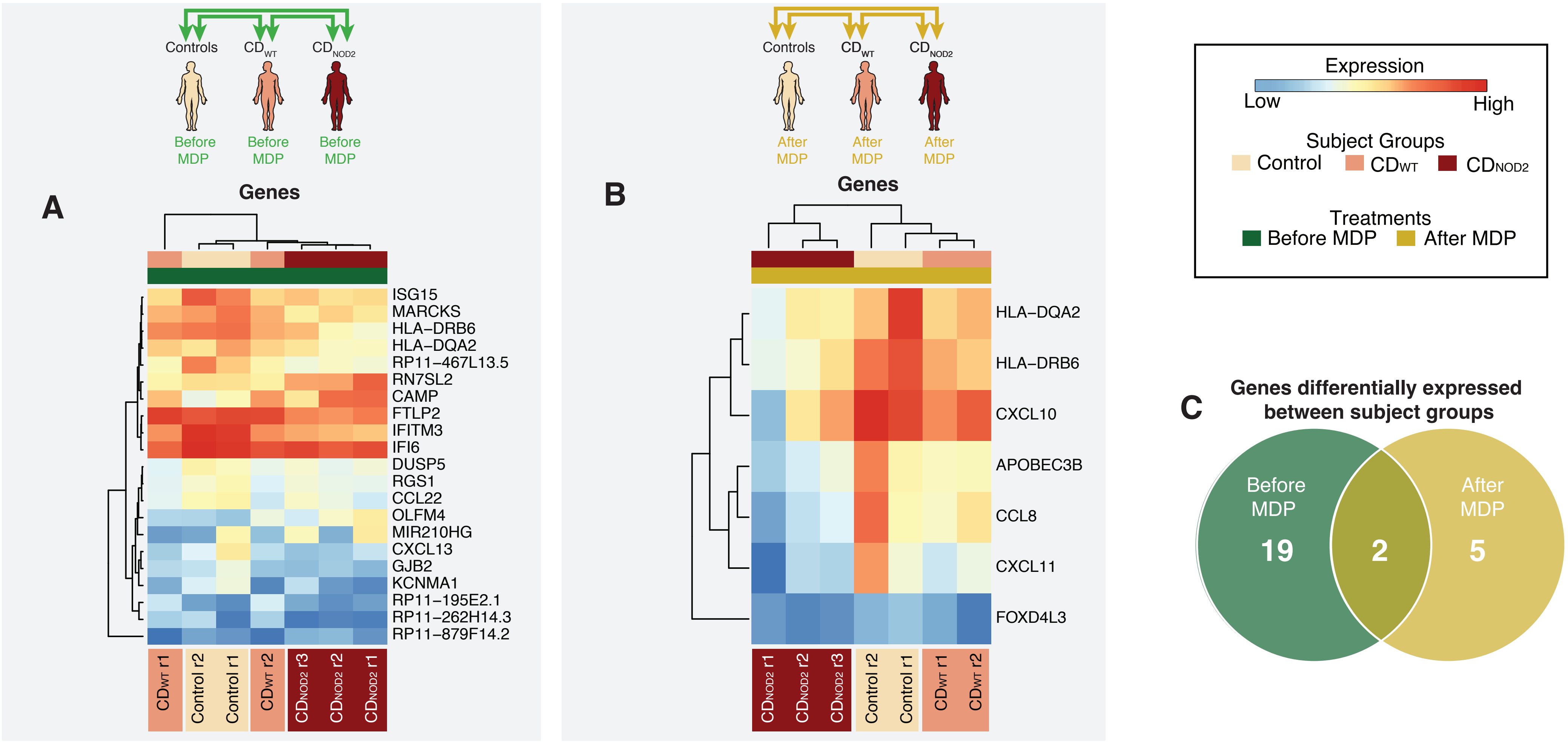

Controls
CDWT
CDNOD2
Before MDP
Before MDP
Before MDP
A
Genes
ISG15
MARCKS
HLA–DRB6
HLA–DQA2
RP11–467L13.5
RN7SL2
CAMP
FTLP2
IFITM3
IFI6
DUSP5
RGS1
CCL22
OLFM4
MIR210HG
CXCL13
GJB2
KCNMA1
RP11–195E2.1
RP11–262H14.3
RP11–879F14.2
CDWT r1
Control r2
Control r1
CDWT r2
CDNOD2 r3
CDNOD2 r2
CDNOD2 r1
Controls
CDWT
CDNOD2
After MDP
After MDP
After MDP
B
Genes
HLA–DQA2
HLA–DRB6
CXCL10
APOBEC3B
CCL8
CXCL11
FOXD4L3
CDNOD2 r1
CDNOD2 r2
CDNOD2 r3
Control r2
Control r1
CDWT r1
CDWT r2
Expression
Low
High
Subject Groups
Control
CDWT
CDNOD2
Treatments
Before MDP
After MDP
C
Genes differentially expressed between subject groups
Before MDP
19
2
After MDP
5

S2

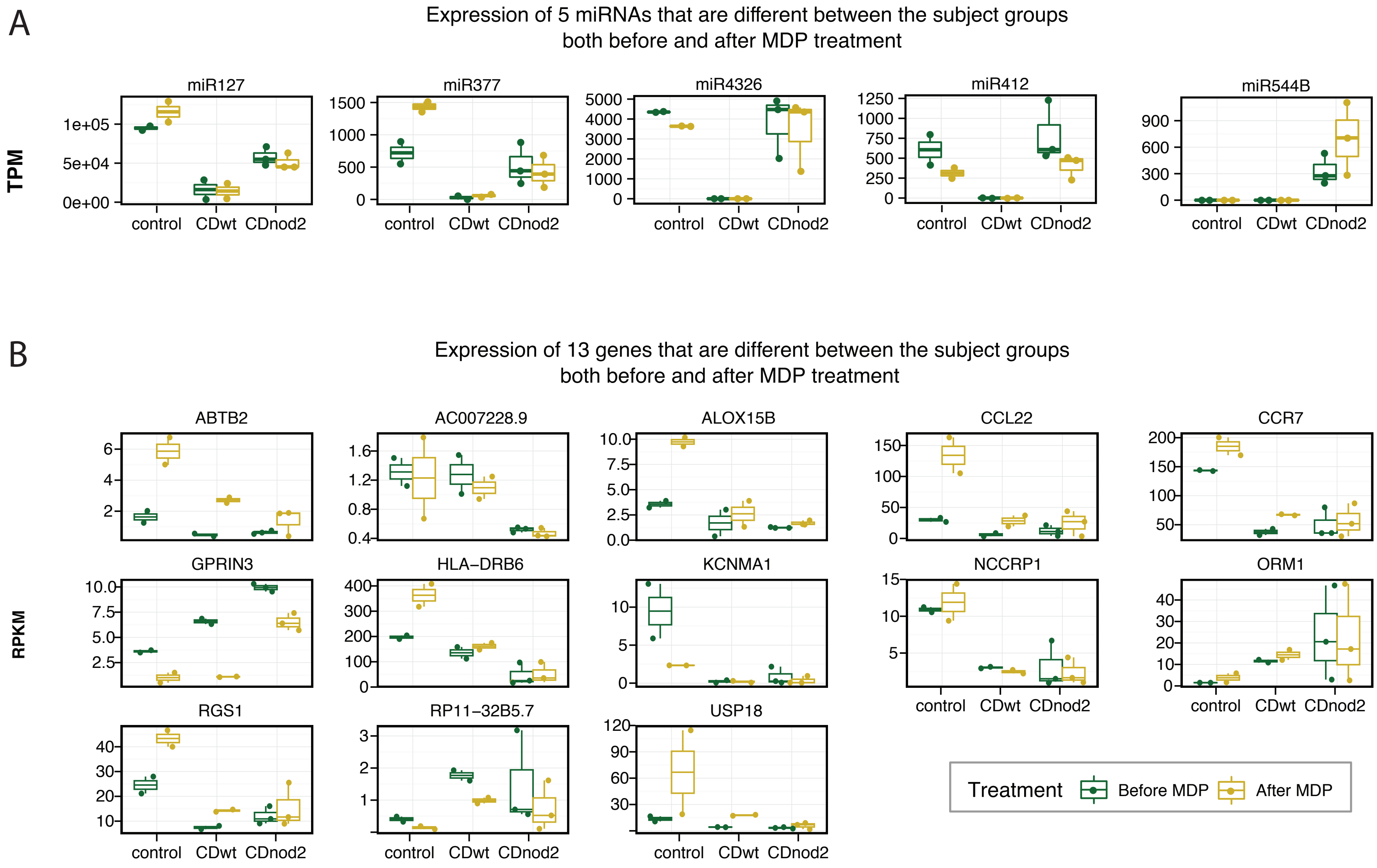
A
Expression of 5 miRNAs that are different between the subject groups
both before and after MDP treatment
TPM
miR127
miR377
miR4326
miR412
miR544B
control
CDwt
CDnod2
B
Expression of 13 genes that are different between the subject groups
both before and after MDP treatment
RPKM
ABTB2
AC007228.9
ALOX15B
CCL22
CCR7
GPRIN3
HLA−DRB6
KCNMA1
NCCRP1
ORM1
RGS1
RP11−32B5.7
USP18
Treatment
Before MDP
After MDP

S3

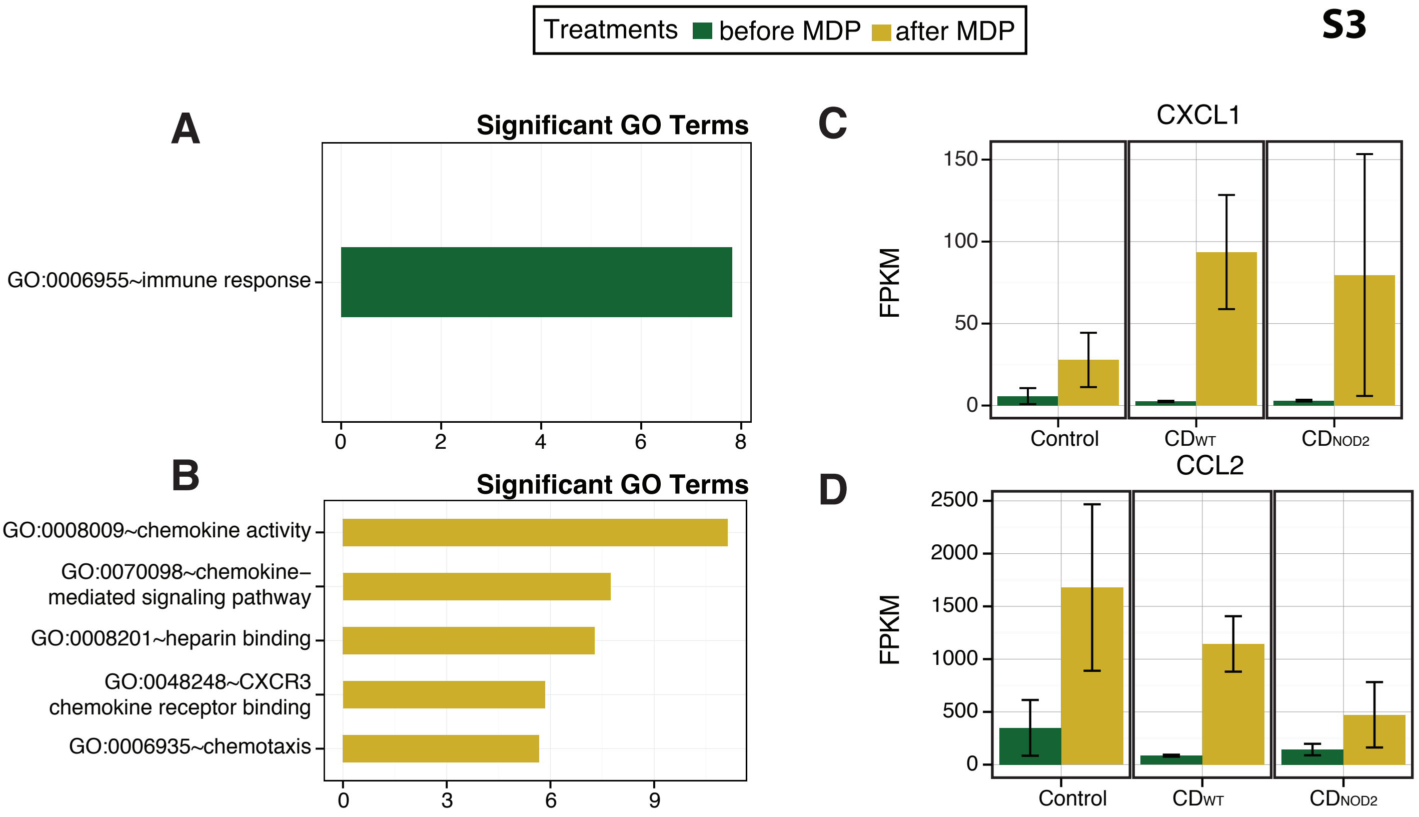

Treatments
before MDP
after MDP
A
Significant GO Terms
GO:0006955~immune response
0
2
4
6
8
B
Significant GO Terms
GO:0008009~chemokine activity
GO:0070098~chemokine–
mediated signaling pathway
GO:0008201~heparin binding
GO:0048248~CXCR3
chemokine receptor binding
GO:0006935~chemotaxis
0
3
6
9
C
CXCL1
FPKM
150
100
50
0
Control
CDWT
CDNOD2
D
CCL2
FPKM
2500
2000
1500
1000
500
0
Control
CDWT
CDNOD2

S4 page1

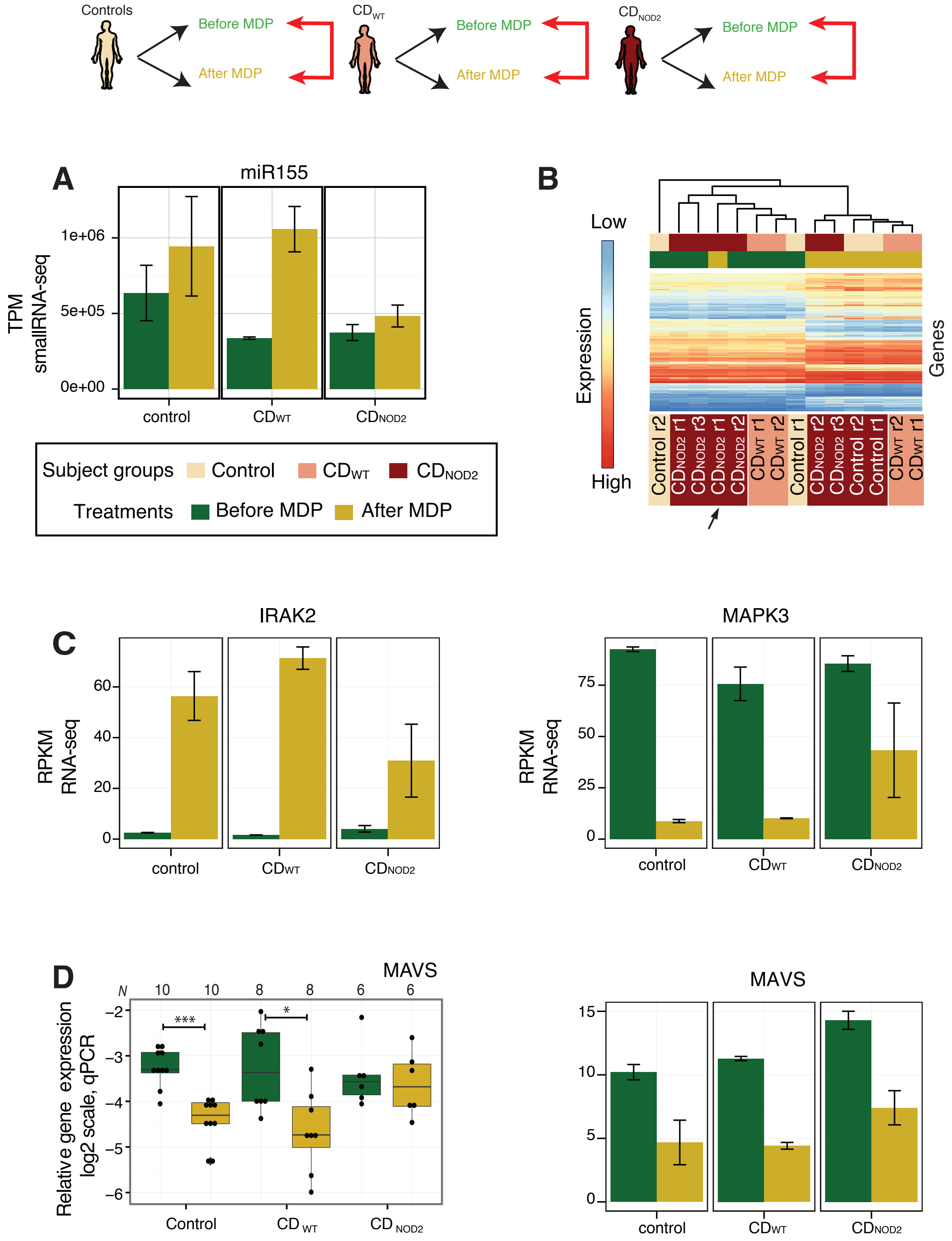

Controls
CD WT
CD NOD2
Before MDP
After MDP
A
miR155
TPM
smallRNA-seq
1e+06
5e+05
0e+00
control
CDWT
CDNOD2
Subject groups
Control
CDWT
CDNOD2
Treatments
Before MDP
After MDP
B
Low
Expression
High
Genes
Control r2
CDNOD2 r1
CDNOD2 r3
CDNOD2 r1
CDNOD2 r2
CDWT r1
CDWT r2
Control r1
CDNOD2 r2
CDNOD2 r3
Control r2
Control r1
CDWT r2
CDWT r1
C
IRAK2
RPKM
RNA-seq
60
40
20
0
control
CDWT
CDNOD2
MAPK3
RPKM
RNA-seq
75
50
25
0
control
CDWT
CDNOD2
D
MAVS
N
10
10
8
8
6
6
Relative gene expression
log2 scale, qPCR
−2
−3
−4
−5
−6
***
*
Control
CD WT
CD NOD2
MAVS
15
10
5
0
control
CDWT
CDNOD2

S4 page2

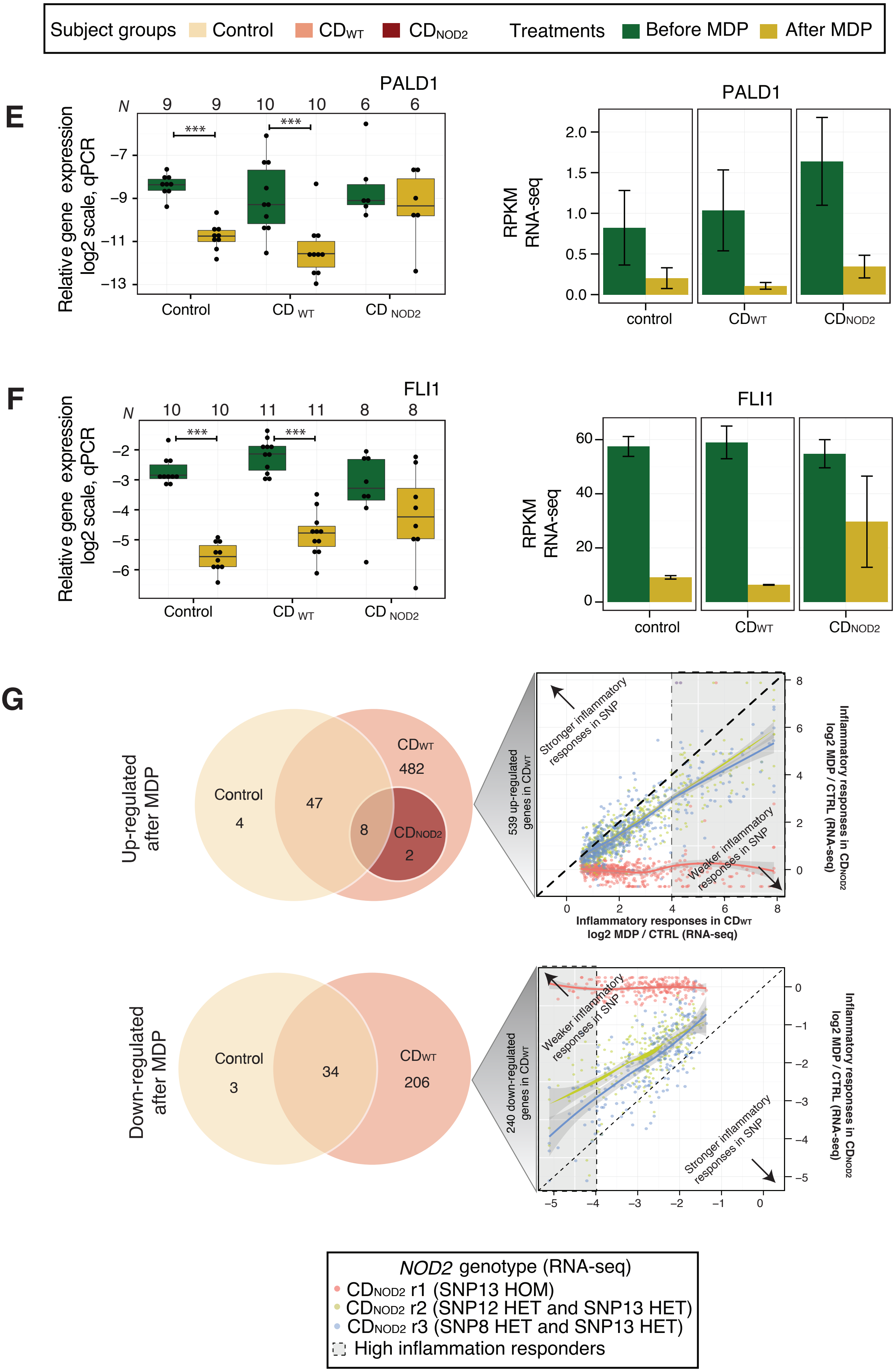

Subject groups Control CDWT CDNOD2 Treatments Before MDP After MDP
E
PALD1
N 9 9 10 10 6 6
***
***
Relative gene expression
log2 scale, qPCR
−7
−9
−11
−13
Control
CD WT
CD NOD2
PALD1
RPKM
RNA-seq
2.0
1.5
1.0
0.5
0.0
control
CDWT
CDNOD2
F
FLI1
N 10 10 11 11 8 8
***
***
Relative gene expression
log2 scale, qPCR
−2
−3
−4
−5
−6
Control
CD WT
CD NOD2
FLI1
RPKM
RNA-seq
60
40
20
0
control
CDWT
CDNOD2
G
Up-regulated after MDP
Control 4
47
8
CDWT 482
CDNOD2 2
539 up-regulated genes in CDWT
Stronger inflammatory responses in SNP
Weaker inflammatory responses in SNP
Inflammatory responses in CDWT
log2 MDP / CTRL (RNA-seq)
Inflammatory responses in CDNOD2
log2 MDP / CTRL (RNA-seq)
Down-regulated after MDP
Control 3
34
CDWT 206
240 down-regulated genes in CDWT
Weaker inflammatory responses in SNP
Stronger inflammatory responses in SNP
NOD2 genotype (RNA-seq)
CDNOD2 r1 (SNP13 HOM)
CDNOD2 r2 (SNP12 HET and SNP13 HET)
CDNOD2 r3 (SNP8 HET and SNP13 HET)
High inflammation responders

S4 page3

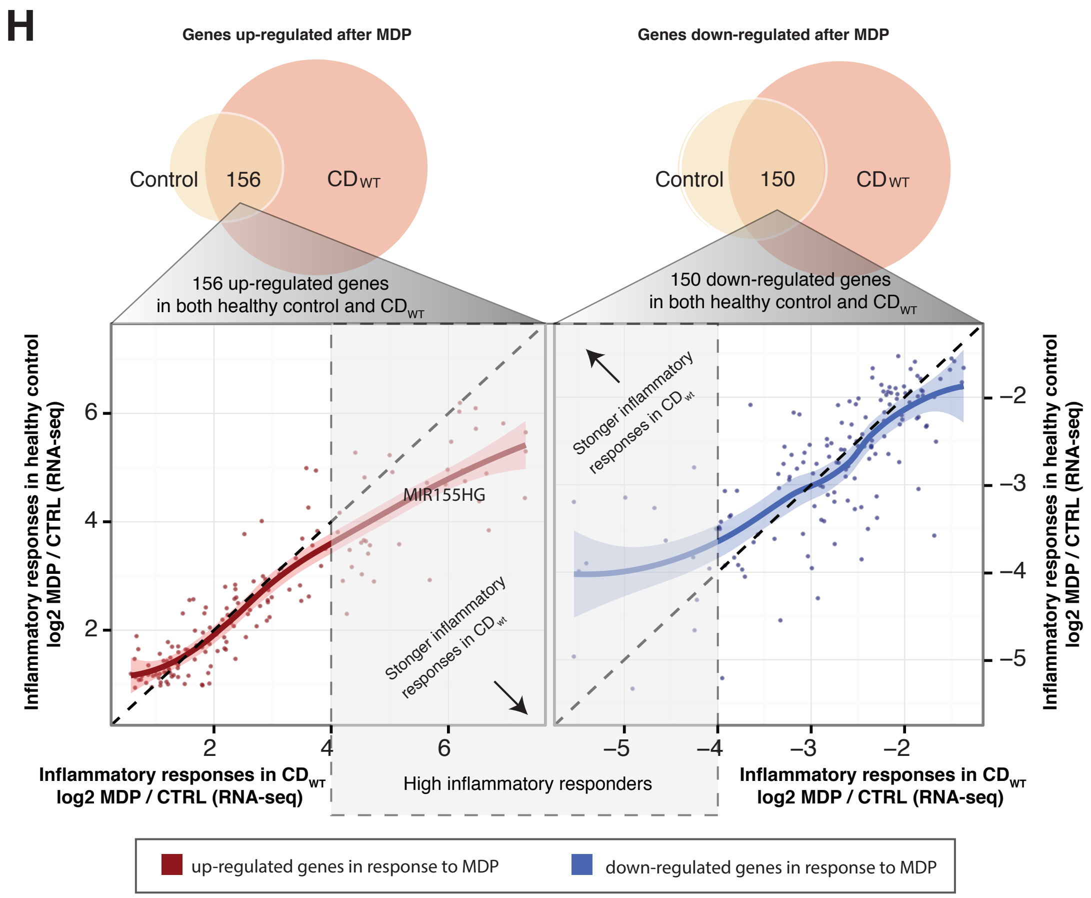
H
Genes up-regulated after MDP
Genes down-regulated after MDP
Control
156
CD WT
Control
150
CD WT
156 up-regulated genes
in both healthy control and CD WT
150 down-regulated genes
in both healthy control and CD WT
MIR155HG
Stonger inflammatory responses in CD wt
Stonger inflammatory responses in CD wt
Inflammatory responses in healthy control log2 MDP / CTRL (RNA-seq)
Inflammatory responses in CD WT log2 MDP / CTRL (RNA-seq)
High inflammatory responders
Inflammatory responses in CD WT log2 MDP / CTRL (RNA-seq)
Inflammatory responses in healthy control log2 MDP / CTRL (RNA-seq)
up-regulated genes in response to MDP
down-regulated genes in response to MDP

S5

A

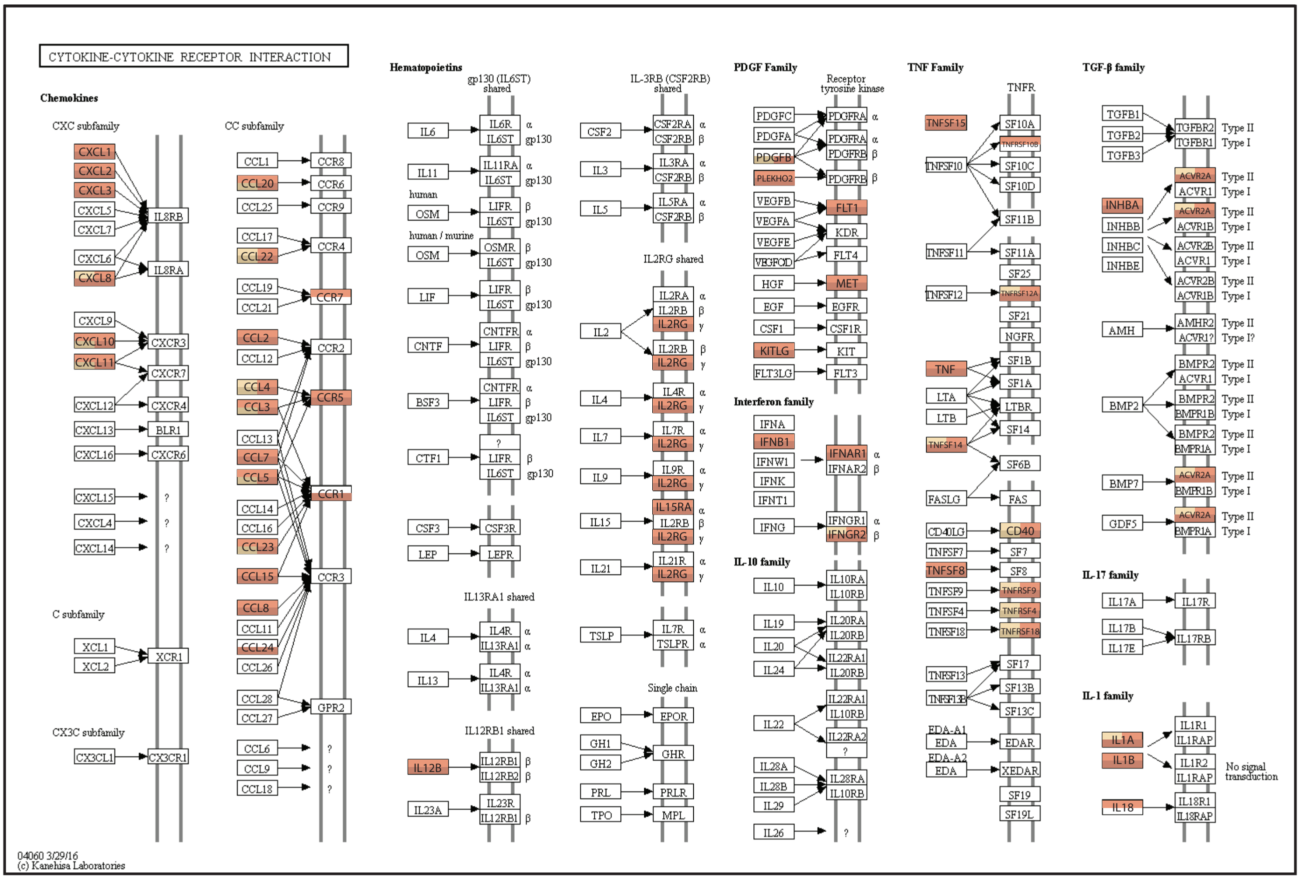
CYTOKINE-CYTOKINE RECEPTOR INTERACTION
Chemokines
CXC subfamily
CC subfamily
C subfamily
CX3C subfamily
Hematopoietins
gp130 (IL6ST) shared
IL-3RB (CSF2RB) shared
IL2RG shared
IL13RA1 shared
IL12RB1 shared
Single chain
PDGF Family
Receptor tyrosine kinase
Interferon family
IL-10 family
TNF Family
TNFR
TGF-β family
IL-17 family
IL-1 family
No signal transduction
04060 3/29/16
(c) Kanehisa Laboratories

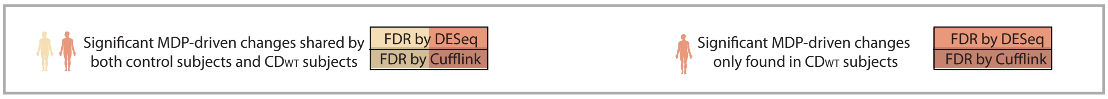
Significant MDP-driven changes shared by both control subjects and CDWT subjects
FDR by DESeq
FDR by Cufflink
Significant MDP-driven changes only found in CDWT subjects
FDR by DESeq
FDR by Cufflink

B

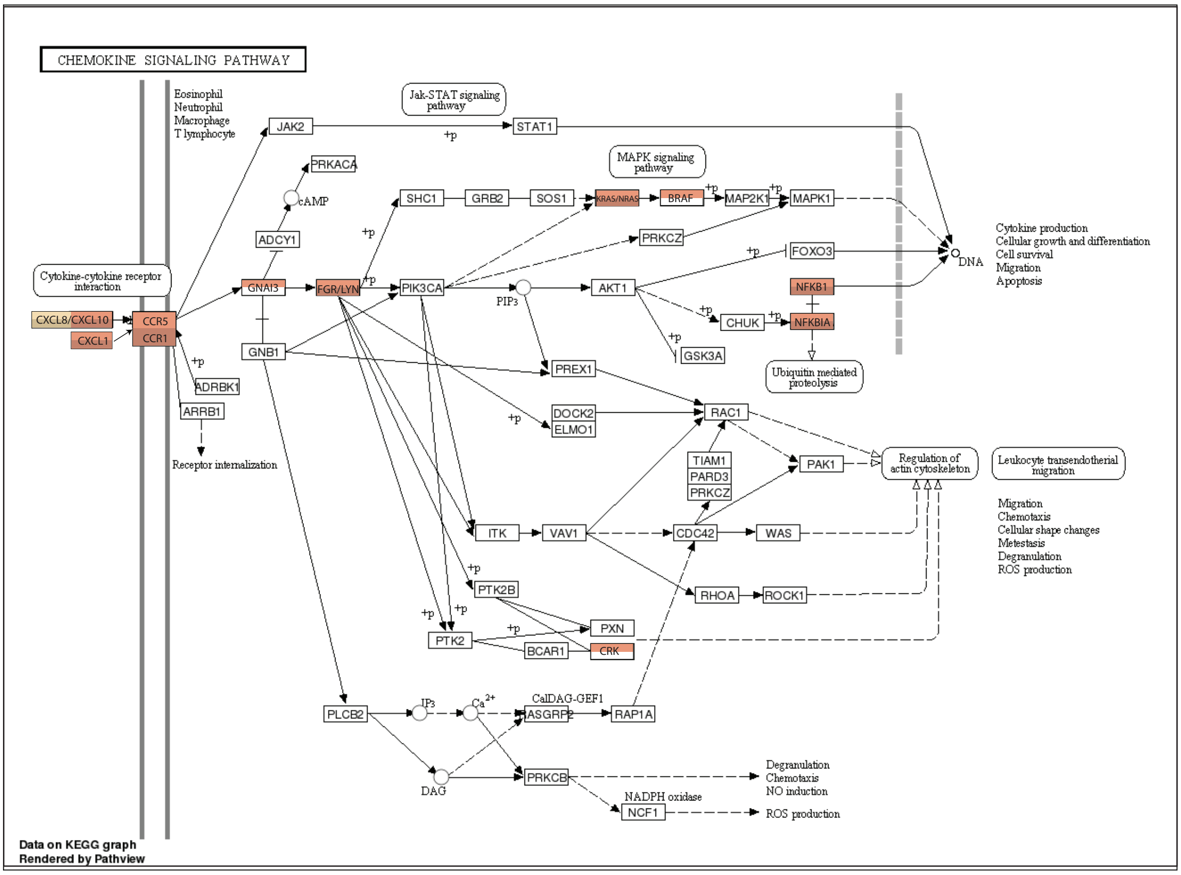
CHEMOKINE SIGNALING PATHWAY
Eosinophil
Neutrophil
Macrophage
T lymphocyte
Jak-STAT signaling pathway
MAPK signaling pathway
Cytokine-cytokine receptor interaction
Receptor internalization
Ubiquitin mediated proteolysis
Regulation of actin cytoskeleton
Leukocyte transendothelial migration
Cytokine production
Cellular growth and differentiation
Cell survival
Migration
Apoptosis
Migration
Chemotaxis
Cellular shape changes
Metestasis
Degranulation
ROS production
Degranulation
Chemotaxis
NO induction
NADPH oxidase
ROS production
CalDAG-GEF1
Data on KEGG graph
Rendered by Pathview

S6

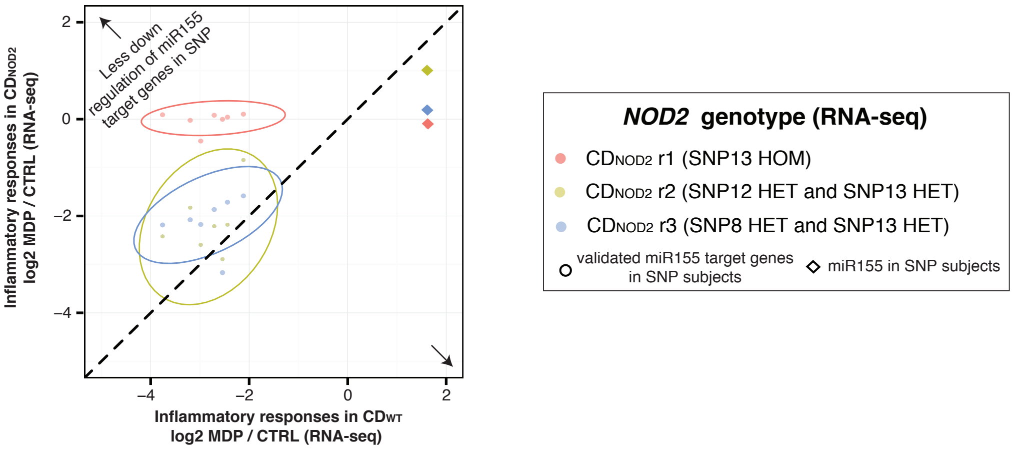
Less down regulation of miR155 target genes in SNP
Inflammatory responses in CDNOD2
log2 MDP / CTRL (RNA-seq)
Inflammatory responses in CDWT
log2 MDP / CTRL (RNA-seq)
2
0
−2
−4
−4
−2
0
2
NOD2 genotype (RNA-seq)
CDNOD2 r1 (SNP13 HOM)
CDNOD2 r2 (SNP12 HET and SNP13 HET)
CDNOD2 r3 (SNP8 HET and SNP13 HET)
validated miR155 target genes in SNP subjects
miR155 in SNP subjects
